# Cracking Shins

**Published:** 1861-12

**Authors:** 


**CRACKING SHINS.**—We remember well in the days of our childhood of hearing our grandmother's kinky-headed little contrabands boasting that it "felt so good," to have their shins cracked with a hard stick. The little monkeys could not exactly explain the thing, that it was the contrasted absence of pain with the immediate, momentary, acute suffering of the stroke. We didn't experiment as we do in later years, as to the doctoring business. We were abundantly content with the "faith" without the "works." Matthew Vasar, the great Poughkeepsie philanthropist, said the other day to a friend, that he "felt like a new man; as if a mountain-weight were lifted from his shoulders," on the occasion of his putting his name to a document which relieved him of the four hundred and eight thousand dollars which he had appropriated to the building, establishment, and support of an Institution in his neighborhood, for the improvement and elevation of the young, and which is in such rapid process of completion, under the energetic, systematic, and judicious management of his old neighbor and friend, Mr. Dubois.

Now, we haven't the least mite of a doubt that a great many of our readers would, at this moment, experience a more beatific sense of "relief" than ever Mr. Vasar did, in being invested with one tenth part of the number of dollars aforesaid, as a free gift. But we are anxious to open up to every honest-hearted reader a source of pleasure quite as soul-delighting as in Mr. Vasar's case; far more ennobling than in the latter; and equally as "striking" as in the matter of shin-bones. It is simply this: take every dollar you can "rake and scrape" without borrowing; do it on the instant; and pay as many debts as it is possible for you to do, beginning with the smallest; and if you get home with every "hole stopped," although you may not have a single mill left, the deliciousness of your happiness, by reason of the contrast between suffering innumerable and distressing duns, and perfect exemption therefrom, will be in exact proportion to the justness, honesty, and goodness of your heart.

